# Emerging Role of microRNAs in Stroke Protection Elicited by Remote Postconditioning

**DOI:** 10.3389/fneur.2021.748709

**Published:** 2021-10-21

**Authors:** Giuseppe Pignataro

**Affiliations:** Division of Pharmacology, Department of Neuroscience, School of Medicine, “Federico II” University of Naples, Naples, Italy

**Keywords:** microRNA, stroke, remote conditioning, tolerance, preconditioning

## Abstract

Remote ischemic conditioning (RIC) represents an innovative and attractive neuroprotective approach in brain ischemia. The purpose of this intervention is to activate endogenous tolerance mechanisms by inflicting a subliminal ischemia injury to the limbs, or to another “remote” region, leading to a protective systemic response against ischemic brain injury. Among the multiple candidates that have been proposed as putative mediators of the protective effect generated by the subthreshold peripheral ischemic insult, it has been hypothesized that microRNAs may play a vital role in the infarct-sparing effect of RIC. The effect of miRNAs can be exploited at different levels: (1) as transducers of protective messages to the brain or (2) as effectors of brain protection. The purpose of the present review is to summarize the most recent evidence supporting the involvement of microRNAs in brain protection elicited by remote conditioning, highlighting potential and pitfalls in their exploitation as diagnostic and therapeutic tools. The understanding of these processes could help provide light on the molecular pathways involved in brain protection for the future development of miRNA-based theranostic agents in stroke.

## Introduction

Ischemic conditioning is a neuroprotective approach able to make the brain more resistant to an ischemic insult through the exposure to a subthreshold stimulus. This method provides neuroprotection when the conditioning stimulus is administered either before or after the detrimental ischemia, i.e., preconditioning or postconditioning.

Indeed, ischemic preconditioning is an endogenous defensive process triggered by a subclinical ischemic event that increases tissue resilience or, in other words, organ resistance to a subsequent, typically dangerous, ischemia episode. Non-ischemic conditioning cues can also promote neuroprotection against an ischemic insult, a phenomenon known as “cross-protection”(1). Surprisingly, when the subliminal boost is delivered after the ischemia insult, the neuroprotection achieved, referred to as postconditioning, is comparable to that shown in ischemic preconditioning models. Remarkably, combining preconditioning and postconditioning does not result in greater protection than either treatment alone (2).

**Figure 1:**
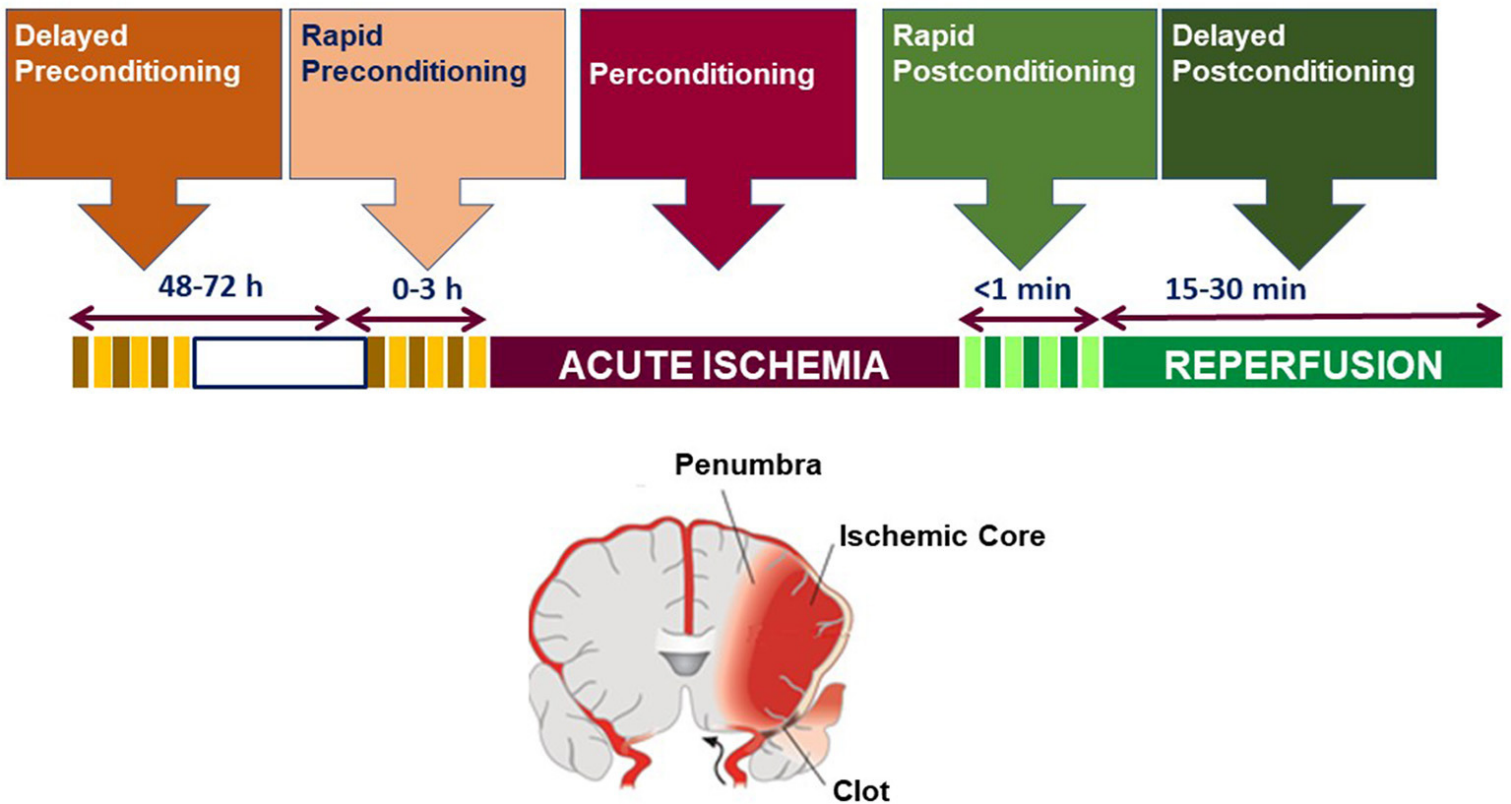
Temporal profile classification of brain conditioning.

We define preconditioning, perconditioning, and postconditioning from a strictly temporal standpoint, depending on whether the conditioning stimulus is delivered before, during, or after the detrimental ischemia (Figure 1).

It is now well recognized that stressing preconditioning or postconditioning stimuli elicit multiple endogenous defensive mechanisms in the brain, resulting in a latent protective phenotype. When the lethal ischemic insult is delivered inside this inactive protective phenotype, a partitioned set of reactions are triggered that are strikingly different from the phenotype of the unprimed or non-preconditioned brain, resulting in the so-called ischemia-tolerant phenotype (3).

Surprisingly enough, in the last years we and others produced data supporting the idea that preconditioning and postconditioning exert their effects also when applied to an anatomical site distant from the brain (4–8). In fact, remote ischemic conditioning (RIC) represents an innovative and attractive protective approach in brain ischemia. This method is designed to elicit the initiation of endogenous tolerance processes by providing a not-dangerous ischemic event in a distant tissue, i.e., arms or limbs, leading to a protective systemic response against stroke. Several studies have examined the effectiveness of RIC in reducing the effects of ischemic brain injury, as well as the potential pathophysiological pathways involved (4–8).

Among the multiple candidates that have been proposed as putative mediators of the protective effect generated by the subthreshold peripheral ischemic insult, it has been hypothesized that also in the case of remote conditioning, as it occurs in the case of direct conditioning (9–13), miRNAs may play a vital role in the infarct sparing effect (5, 7).

The effect of miRNAs during RIC can be exploited at different levels: (a) as transducers of protective messages to the brain or (b) as effectors of brain protection.

This review will summarize the most recent evidence supporting the involvement of microRNAs in the protection of the brain caused by remote conditioning, highlighting the potential and pitfalls in their exploitation as diagnostic and therapeutic tools.

## microRNAs as Therapeutic and Diagnostic Tools

For more than a century, the central view that has dominated molecular biology has been that protein production is mediated by the DNA–RNA–protein axis, which only involves transcription and translation mechanisms to enable decoding of the human genome for production of specific proteins. However, over the recent years, the discovery of non-coding RNAs has radically revolutionized this theory, defining new mechanisms involved in the modulation of protein expression. The human genome project discovered that protein-coding sequences make up only 1.5% of the genome, while introns, regulatory DNA sequences, interspersed elements, and non-coding RNA (ncRNA) molecules make up the remaining 98.5% (14). Without a doubt, the majority of mammalian genomes are translated into ncRNAs, many of which are spliced or processed into smaller products. Short ncRNAs and long ncRNAs are the two types of ncRNAs that have been identified and characterized so far. MicroRNA (miRNA), small interference RNA (siRNA), small nuclear RNA (snRNA), small nucleolar RNA (snoRNA), PIWI-interacting RNA (pi-RNA), transfer RNA (tRNA), circular miRNA (circRNA), ribosomal RNA (rRNA), and other uncharacterized tiny particles are among the short ncRNAs. As of now, miRNAs are characterized as RNA molecules of 18–24 nucleotides in length, transcribed from genes or from intronic regions of other genes, and may promote mRNA degradation or restrict protein translation to control gene expression. More than 60% of mammalian mRNAs are thought to be targeted by at least one miRNA, according to computational predictions (9, 15–19).

Later advances in the topic of miRNAs and their relationship to human diseases have revealed that miRNAs are useful biomarkers and possible disease-modifying agents (20). MiRNA expression profiles exhibit significant changes in response to disease, suggesting that miRNAs are important controllers of disease-related pathways (20).

MiRNAs are considered “downregulators” of gene expression by means of two primary mechanisms: (1) cleavage of mRNA and (2) repression of mRNA translation. MiRNAs interact with mRNA targets through partial sequence complementation, regularly inside the 3′ untranslated region of the mRNA target. In specific, nucleotides 2–7 of the miRNA (beginning from the 5′ end), named the “seed” sequence, are imperative for target binding. The extent of base pairing between miRNA and its target mRNA is currently thought to determine whether the mRNA is degraded or translationally repressed (21, 22).

miRNAs are differentially expressed among tissues, between male and female, during different developmental stages or in response to specific physiological or pathophysiological conditions (17, 23–25). Examples include miR-122, which is preferentially expressed in the liver (24), miR-133a and miR-133b, which are highly enriched in muscle (25), and the miR-302 family members which are specifically expressed in stem cells (26).

### microRNAs as Therapeutic Tools

The development of precise and fast assays for miRNA target identification has played a considerable impact in the study of miRNA roles and in the characterization of the biologic activities in which they are implicated. Since the discovery of the potentialities of miRNAs as therapeutic agents, several effective algorithms have been developed for the prediction of miRNA targets (27–32).

There are various extending endeavors to create therapeutics that straightforwardly can target miRNAs, and agreeing to the sort of miRNA, its expression, and its work, diverse approaches are utilized to overexpress or restrain miRNAs (33).

A miRNA mimic is applied to replace the miRNA concentration, eventually reduced in the progression of a pathologic state. This approach was created in 2007 as gain-of-function instrument for particular miRNAs and comprises engineered double-stranded RNAs that are specifically recognized by RNA-induced silencing complex (RISC) (34). These miRNA-like RNA fragments have a 5′ end that gets a sequence with partial complementarity to the 3′UTR of the target genes, in this way imitating the miRNA working mode. This innovation has been further developed by pharmaceutical companies, and a library of miRNA mimics is accessible for all human miRNAs found until presently. In addition, these particles experience modifications in backbone and ribose to advance steadiness *in vivo* and to overcome impediments related to pharmacodynamics. Over the years, other important changes have been made utilizing adeno-associated infection (AAV) vectors and tissue-specific promoters to improve the tissue specificity impact of miRNA mimics (35).

On the other hand, blocking a miRNA may represent a therapeutic option in some pathological conditions characterized by high levels of expression of those miRNAs involved in deleterious pathways. Anti-miRNAs are nucleotide sequences able to bind to the mature miRNA guide strand, causing its inhibition (20). Several methods are used to add modifications with the intent to generate stable and deliverable miRNA inhibitors, creating a class of antagonists known as antagomirs. Among these chemical modifications are included cholesterol conjugation and the use of locked nucleic acid (LNA) (36). LNA modification increases the stability and nuclease resistance of antisense oligonucleotides and, more importantly, improves the efficiency of hybridization to single-stranded RNA.

Chemically modifying the miRNA using either LNAs or the 2′-O-methyl group (OME) can increase stability *in vivo* and is the basis for many antagomir therapies. Antagomirs are the most frequently used approach for therapeutic use of miRNA therapy (37).

An evolution of anti-miRNAs is represented by miRNA sponges, which are able to inhibit multiple miRNAs simultaneously (38). These molecules can contain a seed sequence for an entire miRNA family, however, they could be also used to target multiple miRNAs.

Finally, the usage of miR-Masks is an emerging strategy for altering miRNA activity (39). These masks are single-stranded 2′-O-methyl-modified antisense oligonucleotides that recognize the miRNA target site on the mRNA 3′UTR and hide it.

### miRNAs as Diagnostic Tools

Beside their expression within tissues and organs, miRNAs are also present outside the cells (40). Several release mechanisms have been hypothesized including release through microvesicles, resulting from outward budding and plasma membrane separation (41). In particular, some vesicles called exosomes, typified by a specific process of biogenesis, are strongly involved in intercellular communication processes and are characterized by the presence of massive amounts of miRNAs inside them (42). Other miRNA transport systems in biological fluids include high-density lipoproteins (HDL) and apoptotic bodies, generated during the programmed cell death process.

Whatever the origins of miRNA, their presence in the blood and the ability to quantify their levels non-invasively cleared the path for the development of peripheral biomarkers for the diagnosis and prognosis of disorders such as brain ischemia. Undoubtedly, miRNA expression levels within the blood are reproducible and demonstrative of different pathologies (43). The interest in peripheral miRNA and their potential application as a biomarker for rapid diagnosis and prognosis are particularly relevant for ischemic patients (44, 45). In fact, several patient-based studies have already reported the occurrence of significant variations in the circulatory expression of miRNAs during cerebral ischemia, correlatable to the entity of the damage (46).

## Translational Relevance of miRNAS in Remote Conditioning Neuroprotection

Over the years, numerous studies demonstrated that stroke may trigger a re-arrangement of the miRNA profile within the cerebral tissue of animals exposed to brain ischemia (47, 48). The first miRNA expression profiling study in cerebral ischemia was performed in 2008, on the entire brains of rats subjected to middle cerebral artery occlusion (MCAO) with reperfusion for 24 or 48 h (49). In this study, it has been shown that 106 transcripts were altered 24 h after stroke induction. However, the number of altered miRNAs was reduced to 82 at 48 h, thus indicating that miRNAs were differentially expressed after brain ischemia in a time-dependent manner. In fact, in the 48-h samples, only rno-miR-99a,−181 (a, b, and c),−195,−328,−379, and−539 were found to be exclusively expressed, whereas, in the 24-h samples, 32 miRNAs (rno-miR-16,−17,−20a,−21,−24,−25,−30a-3p,−34a,−92,−124a,−130a,−132,−134,−151^*^,−210,−215,−324-3p,−322,−329,−342,−361,−374,−382,−383,−422b,−433,−451,−497,−505,−664, let-7d, and let-7f) were found to be exclusively expressed. Notably, rno-miR-206,−214,−223,−290,−292-5p,−298,−327, and−494 were highly upregulated during both ischemia/reperfusion time intervals (49).

These pioneering data were partially confirmed later on in another miRNA profile study carried out in spontaneously hypertensive rats exposed to focal ischemia and reperfusion of different duration (50). In the brain samples obtained from these ischemic animals, it has been found that among the 238 miRNAs examined, 24 miRNAs were upregulated while 22 miRNAs were downregulated at one or more reperfusion time intervals. These data were obtained in the ipsilateral cortex of the ischemic core. Little changes in miRNA expression were detected in the contralateral cortex and in the peri-ischemic cortex. Interestingly, in this study the upregulation of the same five miRNAs identified in the previous work by Jeyaseelan et al., emerged (49), showing the highest upregulation degree at 72 h for miR-206,−214,−223,−290, and−292-5p (49).

Once having established that brain ischemia determines a variation in miRNA expression, the following step was on one side to identify possible miRNAs useful as biomarkers and on another side to identify putative miRNA targets involved in stroke pathophysiology, in order to modulate their expression through miRNAs and to rescue the so-called penumbra region, an area adjacent to the ischemic core, compromised by the ischemic event but not completely damaged.

In this scenario, particular attention was focused to the possibility to restore ionic homeostasis in the penumbra region by using miRNAs to modulate ionic channels and transporters, since the tight correlation between brain damage due to brain ischemia and the disruption of ionic homeostasis is well known (8, 51–53). The plasma membrane sodium/calcium exchanger (NCX1), whose expression is controlled by miR-103 and whose activation ameliorates ischemic damage (51–54), appeared as one of the most promising candidates. Indeed, the capability of AntimiR-103 of inducing a brain-conditioning phenomenon in a rat model of transient brain ischemia has been evidenced (55). The mechanism of action of this LNA anti-miRNA consisted in blocking the detrimental increase of miR-103-1 responsible for the downregulation of NCX1, whose expression is necessary to counteract sodium and calcium imbalance occurring during stroke progression.

Interestingly, more recently, another miRNA, miR-223-5p, emerged as a possible modulator of the K+-dependent Na+/Ca2+ exchanger, NCKX2, a new promising stroke neuroprotective target. In fact, intracerebroventricular infusion of anti-miR-223-5p prevented NCKX2 downregulation occurring after ischemia in rats, thus promoting neuroprotection. Therefore, blocking miR-223-5p by anti-miRNA is a reasonable strategy to reduce the neurodetrimental effect induced by NCKX2 downregulation occurring during brain ischemia (56).

The translatability of miRNAs as biomarkers derives from their physical characteristics. Indeed, as anticipated above, miRNAs are found in a remarkably stable state in human plasma or serum and could be used as biomarkers for a variety of disorders. Chen et al. demonstrated that plasma miRNAs are resistant to RNases as well as other harsh settings such as low/high pH, long-term storage, boiling, and repeated freezing/thawing cycles (57). The levels of miRNA expression in blood have been proven to be repeatable and predictive of illness condition. Although the mechanism of miRNA release into the circulation is unknown, their presence in the bloodstream and relationship with a variety of pathophysiological conditions is now widely established (58) and miRNAs produced by injured or circulating cells are thought to cause enhanced miRNA expression in peripheral biofluids (59).

Many studies demonstrated that this stability is related to the different carriers that mediate miRNA transport, including microvesicles, exosomes, apoptotic bodies, AGO protein complexes to form ribonucleoprotein, and HDL (60, 61). Exosomes, according to current thinking, can regulate the bioactivities of recipient cells by transporting lipids, proteins, and nucleic acids like miRNAs while circulating in the extracellular space, and several studies have shown that exosomes play important roles in immune response, tumor progression, and neurodegenerative disorders.

Taking into account all these considerations and in the attempt to find peripheral markers for stroke, several studies have been conducted to examine expression changes in circulating miRNAs following brain ischemia, at preclinical and clinical levels (10).

Contrasting results have been produced when the expression profiles of miRNAs in the brain were compared to those measured in the blood. In fact, some miRNAs such as miR-290 and miR-494 showed change in their expression in the same direction (upregulation or downregulation) in both tissues at 24 and 48 h, whereas expression levels of some other miRNAs, like miR-150,−195, and−320, exhibited an inverse trend (49). Once again, it should be underlined that brain tissue after ischemic stroke changes over the time, and the per-ischemic region is included in the ischemic core in few hours. miRNA expression is surely influenced by the stage of the disease and the distance from the ischemic core.

Distinct miRNA patterns indicative of the stroke outcome have been found in blood samples of chronic stroke patients within 6–18 months from the stroke onset (62). Interestingly, several miRNAs have shown changes during disease progression. Notably, the number of miRNAs downregulated in all good-outcome stroke samples was usually higher than that of upregulated ones. However, miRNA expression profiles exhibited differential fold change values among the different stroke subtypes; for instance, patients affected by small-artery (SA) stroke show a distinctly different pattern from that of patients affected by large-artery (LA) stroke. In specific, among the highly upregulated miRNAs measured in samples derived from patients affected by SA stroke, seven miRNAs, miR-130b,−29b,−301a,−339-5p,−532-5p,−634, and 886-5p, changed more than two-fold (62).

Recently, miR-107, miR-128b, and miR-153, three brain-enriched miRNAs, were identified within 24 h from hospital admission in plasma samples of patients affected by brain ischemia, demonstrating that their levels were upregulated after stroke onset and positively correlated with the severity of cerebral ischemic injury (63). Interestingly, no correlation was found between age and smoke with the levels of miRNAs, thus suggesting that this upregulation was mediated only by ischemic insult. In parallel, a further study examined the levels of two atherosclerosis-related miRNAs, miR-185, and−146a, in plasma of ischemic stroke patients in the acute phase, 1–5 days, or subacute phase, 6–30 days (10, 37). MiR-185 was found to be downregulated in both the acute and subacute phases, whereas miR-146a was found to be downregulated in the acute phase but upregulated in the subacute phase. Finally, eight miRNAs were found to be differentially expressed in blood collected 28 h after stroke onset (64). In fact, miR-122, miR-148a, let-7i, miR-19a, miR-320d, and miR-4429 were reduced, while miR-363 and miR-487b were upregulated in the bloodstream of acute ischemic patients. These miRNAs were predicted to be regulators of a number of genes and pathways linked to brain ischemia and involved in immune activation, leukocyte extravasation, and thrombus formation.

In the light of these premises and considering the versatility of miRNAs, the identification of pivotal miRNAs may represent a crucial step in defining new putative theranostic tools in stroke. Therefore, the research in the field has been attracted to the possibility of selecting peculiar miRNAs involved in endogenous neuroprotective phenomena including preconditioning and postconditioning.

The idea that ischemic preconditioning (IP) reprograms the response to ischemic injury and determines an altered expression of genes and proteins is well known and commonly accepted (3). This evidence drives researchers to examine whether cerebral ischemic preconditioning might be related to changes within the expression of miRNAs in brain tissue (9, 12, 13, 48). In specific, the impact of IP on miRNA expression profiles was basically assessed by three experimental works. In the first work, only eight miRNAs out of 360 analyzed were selectively upregulated in the brain of rats 3 h after IP (11). These miRNAs were categorized into the following groups: miR-200 family, including miR-200a,−200b,−200c,−429, and−141, and miR-182 family, including miR-182,−183, and−96. Notably, the increased expression of miR-200b,−200c, and−429 could explain the protective upregulation of HIF1α observed in the brain of rats subjected to IP. Indeed, these miRNAs target prolyl hydroxylase two gene (PHD2), an enzyme involved in HIF1α catabolism (11).

In another study, the effect of IP-only ischemia and post-IP tolerance to ischemia on brain miRNA expression profiles was examined; differences in terms of miRNA expression between male and female ischemic mice were also reported (17). In particular, a large subset of miRNAs was uniquely dysregulated in the IP group, including members of the miR-200 and−182 families, which were overexpressed. In addition, *in silico* prediction analysis allowed the identification of target mRNAs, dysregulated following ischemic tolerance. Notably, the predicted target methyl-CpG-binding protein 2 (MeCP2), which is a global regulator of transcription, appeared of particular interest in the development of the ischemic damage, and further confirmatory analysis demonstrated the relationship between miR-132 and MecCP2. Indeed, the overexpression of MeCP2 observed by immunohistochemical staining in brain tissue during ischemic tolerance was accompanied by the reduced expression of miR-132 (16, 17).

MeCP2 emerged as an important miRNA target also in another study carried out in spontaneously hypertensive rats exposed to ischemic preconditioning (50). Among the 265 miRNA screened, only the expression of 20 miRNAs was exclusively modified by preconditioning. In particular, 11 miRNAs were overexpressed, and nine miRNAs showed a reduced expression (16). Beside MeCP2, other major pathways targeted by stroke-regulated miRNAs and participating in cell regulation, proliferation, and apoptosis comprised MAP-kinase, mTOR, Wnt, and GnRh (50).

Few information is available on the role played by miRNAs in remote ischemic limb post-conditioning (RLIP), and studies in this field are in its infancy as testified by the scarcity of published papers. However, since this neuroprotection strategy presupposes a cross talk between the periphery and the central nervous system, it is possible to hypothesize that miRNAs play a role in this process of cell-to-cell communication.

Indeed, RLIP determines a marked neuroprotection by a not dangerous occlusion of the femoral artery (5). Although this method is being evaluated in clinical trials all over the world, the mechanisms activated by RLIP and implicated in the protection have yet to be fully understood (5, 65–67).

Seeing enormous potential, several research groups have focused their efforts on evidence that a short blockage of a distant artery, such as the femoral artery, can protect the brain against ischemic insults, a process known as RIC (4, 5, 65, 68–71).

In a clinical trial published in 2010, this procedure has led to excellent results in patients affected by myocardial infarction, when performed prior to percutaneous coronary intervention (72, 73).

Independently from tissue or organ affected by the harmful event, the mechanisms underlying the phenomenon of RLIP are classified in three steps:

1. The first events occurring in the remote organ or tissue, generated by the RIC stimulus (71). Indeed, blockage of the arm with a tourniquet or blood pressure cuff can trigger the release of autacoids like adenosine, bradykinin, and calcitonin gene-related peptide, or likely of miRNAs, which protect the target organ or tissue (5, 65).
2. The type of the protection signal that is sent from a distant organ to a target organ. Neural and humoral mechanisms have been postulated as means to transport the peripheral signal from an organ such as a leg to a distant organ such as the brain or heart to explain how this phenomenon works (5, 65).
3. The event that takes place in the target organ and mediates the protective response (69).

Since miRNAs represent good candidates involved in all three phases above described, a putative translational strategy to induce stroke neuroprotection could consist in identifying miRNAs involved in these processes and in modulating these identified miRNAs by using either miRNA mimics or miRNA blockers, anti-miRNAs (9, 18, 19). In this regard, the use of miRNA mimics or anti-miRNAs appears to be a very promising method, given that a single miRNA can regulate the expression of multiple proteins at the same time and that cerebral ischemia is a multifactorial disorder with multiple potential therapeutic targets (9, 16, 18, 19).

Recently, microarray microfluidic analysis of 810 miRNAs in the ischemic rat brain revealed that let-7a-5p, miR-143-3p, miR-451-5p, and miR-485-3p had more significant expression alterations than the others. Their levels did increase significantly 24 h after ischemia induction, but when damaging ischemia was followed by remote ischemic postconditioning treatment, they were practically back to pre-ischemic levels (7). Further, more targeted investigations revealed that only let-7a and miR-143, out of the four miRNAs found and selected, would represent an important tool for stroke intervention. These findings are in line with earlier expression data and functional trials, as well as research on putative miRNA targets (49, 50). Indeed, knocking out the let-7a gene can protect against cerebral ischemia/reperfusion injury by reducing apoptosis and inflammatory reaction markers, as evidenced by a decrease in the number of p-p38 MAPK- and p-JNK-immunoreactive cells, as well as lower levels of TNF-α and IL-6 after treatment with a let-7a inhibitor (74). Let-7a has also been shown to target mitogen-activated protein kinase phosphatase 1 (MKP-1), which inactivates JNK1/2 and p38, suggesting a role for this miRNA during neuroinflammation and apoptosis (74).

Many membrane proteins involved in transduction pathways and solute transporters from different families are identified as probable targets of miR-143-3p (75). As a result, its function appears to be linked to cell responses to external stimuli. Recently, miR-143 has been found to play a role in increasing mitochondrial damage in myocardial ischemia *via* targeting PKCε (75). The activation of PKCε in cardiac myocytes is related to the opening of KATP channels. The latter occurs by preventing the opening of the mitochondrial permeability transition pore (mPTP) (75, 76). Notably, it has been demonstrated that a reduction of focal cerebral ischemic injury induced by delayed remote postconditioning may be achieved also through opening of KATP channels (77). Taken together, these data suggest that reduction of miR-143 expression in the brain after tMCAO plus RLIP might prevent PKCε downregulation, with subsequent opening of KATP channels, thus promoting mPTP closure. Although the action of these two miRNAs occurs at the neuronal level, miRNA-143 expression was also seen in the bodies of astrocytic GFAP-positive cells, validating the idea that astrocytes and neurons show different miRNA expression patterns after ischemia (78). Other investigations have corroborated the distinctive expression of miRNA in neurons and glial cells, and numerous hypotheses have been proposed to explain this phenomenon (78, 79). As a result, after remote postconditioning, astrocytes and neurons express different miRNAs; this could be due to differences not only in their miRNA repertoires but also in their cell-specific roles within the CNS.

It is worth noting that let-7a and miR-143 mimic therapy significantly reduces the neuroprotection afforded by RLIP. As a result, these two miRNAs and the proteins they target appear to be potential RLIP-induced neuroprotection mediators. More research is needed to understand which of the target proteins is involved in remote postconditioning-induced neuroprotection. As a result, altering the levels of expression of these miRNAs and their target proteins appears to be a promising stroke therapeutic strategy.

## Exploitation of miRNAs as Stroke Theranostic Tools: Pitfalls and Clinical Perspectives

The incessant work of recent years in studying the role of ncRNAs in stroke made important contributions that advance both the so-called miRNA revolution and our knowledge of the possibilities of exploitation of miRNA as theranostic tools in stroke.

A list of miRNAs responsible for the infarct-sparing effect of brain conditioning is now available (7, 9–13, 17). Furthermore, it is now clear that delivery of a class of the miRNA homologs may represent a novel avenue in therapy, and the changes in miRNA plasma concentrations could be used as a biomarkers. However, the preliminary findings raise significant concerns and debates about their applicability in clinical practice.

To begin, it is critical to note that all conclusions are derived from preclinical models. Several interventions that had been shown to enhance outcomes in experimental animal stroke models, however, failed in clinical studies. Systematic assessments of experimental stroke studies have consistently found low-quality scores, unfavorable publication bias, and a lack of data from female, elderly, or comorbid animals, casting doubt on the robustness and predictive utility of single-laboratory preclinical investigations. The new idea of a multicenter preclinical randomized controlled trial (pRCT) is developing as a critical step before transitioning from animal modeling to clinical trial to improve the translation of therapy efficacy from bench to bedside. Notably, we have recently begun a multicenter preclinical study in rats and mice of both sexes to explore the efficacy of RIC in the experimental model of temporary middle cerebral artery (MCA) occlusion (trial registration number PCTE0000177).

An additional and arguably tangential issue that deserves investigation is the assessment that the majority of papers published in this field did not associate the described favorable effects with transport *via* exosomes, a finding that appears to contradict the emerging concept that extracellular vesicles serve as vectors for the RIPC-initiated humoral communication of protective signals to the brain.

The cellular source of the circulating miRNAs released in response to the RIPC stimulation is a third question that will be difficult to answer: are miRNAs released from skeletal muscle or from a more conventional, blood-borne source?

Fourth is the importance of defining a temporal profile of expression. miRNAs are released in response to a stimulus in a time-related manner. Defining a precise timing of determination after stroke onset is fundamental in the case miRNAs are used as biomarkers, but is also extremely important when miRNA mimics are used as therapeutic tools.

In the vast majority of preclinical studies, MCAO was used to confirm alterations in specific miRNAs in young healthy male rats or mice. Furthermore, the majority of clinical stroke studies did not specify the number of male and female patients who participated in the trials, making it difficult to discuss the importance of gender-specific changes in miRNAs and their contributions to ischemic stroke and/or responses to antagomirs or mimics. In future investigations, men and females animals must be used to confirm the upregulation or downregulation of particular miRNAs. Finally, because most stroke patients have comorbidities and are over the age of 50, animal stroke models with hypertension, hyperlipidemia, and diabetes mellitus, as well as older animals, should be included in confirmation investigations.

Lastly, it will be extremely important to define a delivery system. A successful miRNA therapy necessitates a precise and effective delivery method. To address the intrinsic instability of miRNA in circulation, off-target effects, and improper distribution, a delivery method is required, but it does not need to reduce cell permeability, increase excretion, or accumulate in off-target organs (10). MiRNAs can be injected intravenously or subcutaneously because they are small and water-soluble. Because miRNAs are single-stranded and open-ended, they are either destroyed by systemic nucleases in circulation or eliminated by the kidneys (56).

Accumulation in non-target organs such as liver and spleen, non-specific absorption, excretion, toxicity, and an immune-mediated response are among issues that might arise with various delivery systems (56). Systemic delivery must consider the delivery system's stability in circulation as well as tissue-specific targeting. Some options for systemic delivery have been developed through ongoing research. In this regard, the viral capsid can be modified for tissue-specific delivery, and viral vectors can be used to boost circulatory stability during translocation (56). Adeno-associated viruses have exhibited great tissue specificity and acceptable safety profiles in clinical trials for gene therapy (37). Viral vectors, however, do have pitfalls including immune reactions and viral integration into the host genome (56). Other lipid-based vectors, like liposomes, are protective against nuclease, lysosomal, and endosomal degradation and can be effectively used (40). As natural transporters, exosome delivery systems are an attractive option. These offer specificity by binding in a receptor-mediated fashion, limiting off-target side effects (59). Nanoparticles are another possible strategy and have been used in an attempt to overcome excessive inflammatory reactions (8). An intriguing concept for drug delivery for IR injury is “passive drug targeting” where a colloid-based drug delivery system is utilized. Because the endothelium at these places is weakened, these chemicals collect at sites of inflammation, allowing passive diffusion through the artery (40). Another option for systemic administration is mesenchymal stem cell-derived extracellular vesicles (MSC-EV), which have been investigated *in vivo* and found to be able to transport miRNAs (52). MSC-EV, like the other delivery systems, are currently being studied for clinical usage, with promising results.

## Conclusions

Efforts will be needed in the next decade of miRNA research to enhance and evolve the tools for miRNA analysis and validation, as these technologies will be critical in establishing direct correlations between miRNA-mediated post-transcriptional gene expression and disease (9, 19). Furthermore, it is important to underline the need to speed up all those technical procedures capable of detecting miRNAs in the shortest and simplest possible way, to avoid running into the same problems currently present with the use of CT and MRI to make differentiated diagnosis of ischemic and hemorrhagic stroke.

## Author Contributions

The author confirms being the sole contributor of this work and has approved it for publication.

## Funding

This work was supported by grants from Programma Operativo Nazionale PON NEON (ARS01_00769) from the Italian Ministry of Research, MIUR, to GP.

## Conflict of Interest

The author declares that the research was conducted in the absence of any commercial or financial relationships that could be construed as a potential conflict of interest.

## Publisher's Note

All claims expressed in this article are solely those of the authors and do not necessarily represent those of their affiliated organizations, or those of the publisher, the editors and the reviewers. Any product that may be evaluated in this article, or claim that may be made by its manufacturer, is not guaranteed or endorsed by the publisher.

## References

[1] Gidday JM. Cerebral preconditioning and ischaemic tolerance. Nat Rev Neurosci. (2006) 7:437iNeur 10.1038/nrn1927 16715053

[2] Pignataro G Meller R Inoue K Ordonez AN Ashley MD Xiong Z. *In vivo* and in *vitro* characterization of a novel neuroprotective strategy for stroke: ischemic postconditioning. J Cereb Blood Flow Metab. (2008) 28:232low M 10.1038/sj.jcbfm.9600559 17882162

[3] Stenzel-Poore MP Stevens SL Xiong Z Lessov NS Harrington CA Mori MA. Effect of ischaemic preconditioning on genomic response to cerebral ischaemia: similarity to neuroprotective strategies in hibernation and hypoxia-tolerant states. Lancet. (2003) 362:1028–0282 10.1016/S0140-6736(03)14412-1 14522533

[4] Pignataro G Esposito E Sirabella R Vinciguerra A Cuomo O Di Renzo G. nNOS and p-ERK involvement in the neuroprotection exerted by remote postconditioning in rats subjected to transient middle cerebral artery occlusion. Neurobiol Dis. (2013) 54:105l Dis 10.1016/j.nbd.2013.02.008 23454199

[5] Hess DC Blauenfeldt RA Andersen G Hougaard KD Hoda MN Ding Y. Remote ischaemic conditioning-a new paradigm of self-protection in the brain. Nat Rev Neurol. (2015) 11:698 Neuro 10.1038/nrneurol.2015.223 26585977

[6] Valsecchi V Laudati G Cuomo O Sirabella R Annunziato L Pignataro G. The hypoxia sensitive metal transcription factor MTF-1 activates NCX1 brain promoter and participates in remote postconditioning neuroprotection in stroke. Cell Death Dis. (2021) 12:423. 10.1038/s41419-021-03705-9 33931586 PMC8087832

[7] Vinciguerra P Cepparulo S Anzilotti O Cuomo V Valsecchi S Amoroso L. Remote postconditioning ameliorates stroke damage by preventing let-7a and miR-143 up-regulation. Theranostics. (2020) 10:12174–88. 10.7150/thno.48135 33204336 PMC7667695

[8] Pignataro G Brancaccio P Laudati G Valsecchi V Anzilotti S Casamassa A. Sodium/calcium exchanger as main effector of endogenous neuroprotection elicited by ischemic tolerance. Cell Calcium. (2020) 87:102183. 10.1016/j.ceca.2020.102183 32120196

[9] Saugstad JA. Non-coding RNAs in stroke and neuroprotection. Front Neurol. (2015) 6:50. 10.3389/fneur.2015.00050 25821444 PMC4358219

[10] Li G Morris-Blanco KC Lopez MS Yang T Zhao H Vemuganti R. Impact of microRNAs on ischemic stroke: from pre- to post-disease. Prog Neurobiol. (2018) 163–164:59–78. 10.1016/j.pneurobio.2017.08.002 28842356 PMC11884751

[11] Lee ST Chu K Jung KH Yoon HJ Jeon D Kang KM. MicroRNAs induced during ischemic preconditioning. Stroke. (2010) 41:1646–646:10.1161/STROKEAHA.110.579649 20576953

[12] Jimenez-Mateos EM. Role of microRNAs in innate neuroprotection mechanisms due to preconditioning of the brain. Front Neurosci. (2015) 21:18. 10.3389/fnins.2015.00118 25954143 PMC4404827

[13] Bell JD Cho JE Giffard RG. MicroRNA changes in preconditioning-induced neuroprotection. Transl Stroke Res. (2017) 8:585. 10.1007/s12975-017-0547-1 28646450 PMC5701644

[14] Naidoo N Pawitan Y Soong R Cooper DN Ku CS. Human genetics and genomics a decade after the release of the draft sequence of the human genome. Hum Genomics. (2011) 5:577 omics 10.1186/1479-7364-5-6-577 22155605 PMC3525251

[15] Ceman S Saugstad J. MicroRNAs: meta-controllers of gene expression in synaptic activity emerge as genetic and diagnostic markers of human disease. Pharmacol Ther. (2011) 130:26l The 10.1016/j.pharmthera.2011.01.004 21256154 PMC3043141

[16] Lusardi TA Farr CD Faulkner CL Pignataro G Yang T Lan J. Ischemic preconditioning regulates expression of microRNAs and a predicted target, MeCP2, in mouse cortex. J Cereb Blood Flow Metab. (2010) 30:744–56. 10.1038/jcbfm.2009.253 20010955 PMC2935903

[17] Lusardi TA Murphy SJ Phillips JI Chen Y Davis CM Young JM. MicroRNA responses to focal cerebral ischemia in male and female mouse brain. Front Mol Neurosci. (2014) 7:11. 10.3389/fnmol.2014.00011 24574964 PMC3920114

[18] Saugstad JA. MicroRNAs as effectors of brain function with roles in ischemia and injury, neuroprotection, and neurodegeneration. J Cereb Blood Flow Metab. (2010) 30:1564–76. 10.1038/jcbfm.2010.101 20606686 PMC2932764

[19] Saugstad JA. MicroRNAs as effectors of brain function. Stroke. (2013) 44:S17–9. 10.1161/STROKEAHA.113.000985 23709715 PMC3740953

[20] Basak I Patil KS Alves G Larsen JP Moller SG. microRNAs as neuroregulators, biomarkers and therapeutic agents in neurodegenerative diseases. Cell Mol Life Sci. (2016) 73:811 iScif 10.1007/s00018-015-2093-x 26608596 PMC11108480

[21] Fabian MR Sonenberg N Filipowicz W. Regulation of mRNA translation and stability by microRNAs. Annu Rev Biochem. (2010) 79:351 chemc 10.1146/annurev-biochem-060308-103103 20533884

[22] Fabian MR Sundermeier TR Sonenberg N. Understanding how miRNAs post-transcriptionally regulate gene expression. Prog Mol Subcell Biol. (2010) 50:1 l Bio 10.1007/978-3-642-03103-8_1 19841878

[23] Tagliaferri S Cepparulo P Vinciguerra A Campanile M Esposito G Maruotti GM. miR-16-5p, miR-103-3p, and miR-27b-3p as Early peripheral biomarkers of fetal growth restriction. Front Pediatr. (2021) 9:611112. 10.3389/fped.2021.611112 33777862 PMC7991078

[24] Lagos-Quintana M Rauhut R Yalcin A Meyer J Lendeckel W Tuschl T. Identification of tissue-specific microRNAs from mouse. Curr Biol. (2002) 12:735 lifi 10.1016/S0960-9822(02)00809-6 12007417

[25] Sempere LF Freemantle S Pitha-Rowe I Moss E Dmitrovsky E Ambros V. Expression profiling of mammalian microRNAs uncovers a subset of brain-expressed microRNAs with possible roles in murine and human neuronal differentiation. Genome Biol. (2004) 5:R13. 10.1186/gb-2004-5-3-r13 15003116 PMC395763

[26] del Jesus AB Lucena-Aguilar G Menendez P. The miR-302-367 cluster as a potential stemness regulator in ESCs. Cell Cycle. (2009) 8:394–8. 10.4161/cc.8.3.7554 19176999

[27] Huang Y Zou Q Song H Song F Wang L Zhang G. A study of miRNAs targets prediction and experimental validation. Protein Cell. (2010) 1:979 Cell 10.1007/s13238-010-0129-4 21153515 PMC4875151

[28] Wang P Li Q Sun N Gao Y Liu JS Deng K. MiRACLe: an individual-specific approach to improve microRNA-target prediction based on a random contact model. Brief Bioinform. (2021) 22:bbaa117. 10.1093/bib/bbaa117 34020537

[29] Zheng X Chen L Li X Zhang Y Xu S Huang X. Prediction of miRNA targets by learning from interaction sequences. PLoS ONE. (2020) 15:e0232578. 10.1371/journal.pone.0232578 32369518 PMC7199961

[30] Wong L You ZH Guo ZH Yi HC Chen ZH Cao MY. MIPDH: a novel computational model for predicting microRNA-mRNA interactions by deepwalk on a heterogeneous network. ACS Omega. (2020) 5:17022–32. 10.1021/acsomega.9b04195 32715187 PMC7376568

[31] Jiang H Yang M Chen X Li M Li Y Wang J. miRTMC: a miRNA target prediction method based on matrix completion algorithm. IEEE J Biomed Health Inform. (2020) 24:3630–630:10.1109/JBHI.2020.2987034 32287029

[32] Jiang H Wang J Li M Lan W Wu FX Pan Y. miRTRS: a recommendation algorithm for predicting miRNA targets. IEEE/ACM Trans Comput Biol Bioinform. (2020) 17:1032–032:10.1109/TCBB.2018.2873299 30281478

[33] Hammond SM. An overview of microRNAs. Adv Drug Deliv Rev. (2015) 87:3–14. 10.1016/j.addr.2015.05.001 25979468 PMC4504744

[34] Wang HW Noland C Siridechadilok B Taylor DW Ma E Felderer K. Structural insights into RNA processing by the human RISC-loading complex. Nat Struct Mol Biol. (2009) 16:1148–148:10.1038/nsmb.1673 19820710 PMC2845538

[35] van Rooij E Kauppinen S. Development of microRNA therapeutics is coming of age. EMBO Mol Med. (2014) 6:851l Med 10.15252/emmm.201100899 24935956 PMC4119351

[36] Elmen J Lindow M Schutz S Lawrence M Petri A Obad S. LNA-mediated microRNA silencing in non-human primates. Nature. (2008) 452:896imat 10.1038/nature06783 18368051

[37] Li Y Mao L Gao Y Baral S Zhou Y Hu B. MicroRNA-107 contributes to post-stroke angiogenesis by targeting Dicer-1. Sci Rep. (2015) 5:13316. 10.1038/srep13316 26294080 PMC4543985

[38] Ebert MR Neilson JR Sharp PA. MicroRNA sponges: competitive inhibitors of small RNAs in mammalian cells. Nat Methods. (2007) 4:721–6. 10.1038/nmeth1079 17694064 PMC3857099

[39] Wang Z. The principles of MiRNA-masking antisense oligonucleotides technology. Methods. Mol Biol. (2011) 676:43–9. 10.1007/978-1-60761-863-8_3 20931388

[40] Weber JA Baxter DH Zhang S Huang DY Huang KH Lee MJ. The microRNA spectrum in 12 body fluids. Clin Chem. (2010) 56:1733–41. 10.1373/clinchem.2010.147405 20847327 PMC4846276

[41] Colombo M Raposo G Thery C. Biogenesis, secretion, and intercellular interactions of exosomes and other extracellular vesicles. Annu Rev Cell Dev Biol. (2014) 30:255l Dev 10.1146/annurev-cellbio-101512-122326 25288114

[42] Basso M Bonetto V. Extracellular vesicles and a novel form of communication in the brain. Front Neurosci.(2016) 10:127. 10.3389/fnins.2016.00127 27065789 PMC4814526

[43] Viswambharan V Thanseem I Vasu MM Poovathinal SA Anitha A. miRNAs as biomarkers of neurodegenerative disorders. Biomark Med. (2017) 11:151Medas 10.2217/bmm-2016-0242 28125293

[44] Bejleri J Jirstrom E Donovan P Williams DJ Pfeiffer S. Diagnostic and prognostic circulating microrna in acute stroke: a systematic and bioinformatic analysis of current evidence. J Stroke. (2021) 23:162e of 10.5853/jos.2020.05085 34102753 PMC8189849

[45] Khoshnam SE Winlow W Farbood Y Moghaddam HF Farzaneh M. Emerging roles of microRNAs in ischemic stroke: as possible therapeutic agents. J Stroke. (2017) 19:166ging 10.5853/jos.2016.01368 28480877 PMC5466283

[46] Giordano M Ciarambino T D'Amico M Trotta MC Di Sette AM Marfella R. Circulating MiRNA-195-5p and−451a in transient and acute ischemic stroke patients in an emergency department. J Clin Med. (2019) 8:130. 10.3390/jcm8020130 30678250 PMC6406765

[47] Vemuganti R. All's well that transcribes well: non-coding RNAs and post-stroke brain damage. Neurochem Int. (2013) 63:438–49. 10.1016/j.neuint.2013.07.014 23954844 PMC3805745

[48] Koutsis G Siasos G Spengos K. The emerging role of microRNA in stroke. Curr Top Med Chem. (2013) 13:1573–573:10.2174/15680266113139990106 23745809

[49] Jeyaseelan K Lim KY Armugam A. MicroRNA expression in the blood and brain of rats subjected to transient focal ischemia by middle cerebral artery occlusion. Stroke. (2008) 39:959oRNA 10.1161/STROKEAHA.107.500736 18258830

[50] Dharap A Bowen K Place R Li LC Vemuganti R. Transient focal ischemia induces extensive temporal changes in rat cerebral microRNAome. J Cereb Blood Flow Metab. (2009) 29:675low M 10.1038/jcbfm.2008.157 19142192 PMC2743462

[51] Pignataro G Sirabella R Anzilotti S Di Renzo G Annunziato L. Does Na(+)/Ca(2)(+) exchanger, NCX, represent a new druggable target in stroke intervention?. Transl Stroke Res. (2014) 5:145–55. 10.1007/s12975-013-0308-8 24323727

[52] Pignataro G Cuomo O Vinciguerra A Sirabella R Esposito E Boscia F. NCX as a key player in the neuroprotection exerted by ischemic preconditioning and postconditioning. Adv Exp Med Biol. (2013) 961:223iolli 10.1007/978-1-4614-4756-6_19 23224883

[53] Annunziato L Boscia F Pignataro G. Ionic transporter activity in astrocytes, microglia, and oligodendrocytes during brain ischemia. J Cereb Blood Flow Metab. (2013) 33:969low M 10.1038/jcbfm.2013.44 23549380 PMC3705429

[54] Maiolino M Castaldo P Lariccia V Piccirillo S Amoroso S Magi S. Essential role of the Na+-Ca2+ exchanger (NCX) in glutamate-enhanced cell survival in cardiac cells exposed to hypoxia/reoxygenation. Sci Rep. (2017) 7:13073. 10.1038/s41598-017-13478-x 29026150 PMC5638850

[55] Vinciguerra A Formisano L Cerullo P Guida N Cuomo O Esposito A. MicroRNA-103-1 selectively downregulates brain NCX1 and its inhibition by anti-miRNA ameliorates stroke damage and neurological deficits. Mol Ther. (2014) 22:1829–38. 10.1038/mt.2014.113 24954474 PMC4428397

[56] Cuomo O Cepparulo P Anzilotti S Serani A Sirabella R Brancaccio P. Anti-miR-223-5p ameliorates ischemic damage and improves neurological function by preventing nckx2 downregulation after ischemia in rats. Mol Ther Nucleic Acids. (2019) 18:1063–71. 10.1016/j.omtn.2019.10.022 31791013 PMC6906731

[57] Chen X Ba Y Ma L Cai X Yin Y Wang K. Characterization of microRNAs in serum: a novel class of biomarkers for diagnosis of cancer and other diseases. Cell Res. (2008) 18:997Y1006. 10.1038/cr.2008.282 18766170

[58] Li WY Jin J Chen J Guo Y Tang J. Tan S. Circulating microRNAs as potential non-invasive biomarkers for the early detection of hypertension-related stroke. J Hum Hypertens. (2014) 28:288serte 10.1038/jhh.2013.94 24132136

[59] Mayr M Zampetaki A Kiechl S. MicroRNA biomarkers for failing hearts?. Eur Heart J. (2013) 34:2782–782 10.1093/eurheartj/eht261 23886919

[60] Makarova JA Shkurnikov MU Turchinovich AA Tonevitsky AG Grigoriev AI. Circulating microRNAs. Biochemistry. (2015) 80:1117–26. 10.1134/S0006297915090035 26555465

[61] Zhang J Li S Li L Li M Guo C Yao J. Exosome and exosomal microRNA: trafficking, sorting, and function. Genomics Proteomics Bioinformatics. (2015) 13:17cs Pr 10.1016/j.gpb.2015.02.001 25724326 PMC4411500

[62] Tan KS Armugam A Sepramaniam S Lim KY Setyowati KD Wang CW. Expression profile of MicroRNAs in young stroke patients. PLoS ONE. (2009) 4:e7689. 10.1371/journal.pone.0007689 19888324 PMC2765616

[63] Yang S Zhao J Chen Y Lei M. Biomarkers associated with ischemic stroke in diabetes mellitus patients. Cardiovasc Toxicol. (2016) 16:213sc To 10.1007/s12012-015-9329-8 26175178

[64] Jickling GC Ander BP Zhan X Noblett D Stamova B Liu D. MicroRNA expression in peripheral blood cells following acute ischemic stroke and their predicted gene targets. PLoS ONE. (2014) 9:e99283. 10.1371/journal.pone.0099283 24911610 PMC4050059

[65] Hess DC Khan MB Hoda N Morgan JC. Remote ischemic conditioning: a treatment for vascular cognitive impairment. Brain Circ. (2015) 1:133irc 10.4103/2394-8108.172885 30221201 PMC6135530

[66] Khan MB Hoda MN Vaibhav K Giri S Wang P Waller JL. Remote ischemic postconditioning: harnessing endogenous protection in a murine model of vascular cognitive impairment. Transl Stroke Res. (2015) 6:69–77. 10.1007/s12975-014-0374-6 25351177 PMC4297613

[67] Landman T Schoon Y Warle M de Leeuw F-E Thijssen D. The effect of repeated remote ischemic postconditioning on infarct size in patients with an ischemic stroke (REPOST): study protocol for a randomized clinical trial. Trials. (2019) 20:167. 10.1186/s13063-019-3264-0 30876432 PMC6419836

[68] Zhao H Ren C Chen X Shen J. From rapid to delayed and remote postconditioning: the evolving concept of ischemic postconditioning in brain ischemia. Curr Drug Targets. (2012) 13:173rgets 10.2174/138945012799201621 22204317 PMC3346695

[69] Hausenloy DJ Yellon DM. Ischaemic conditioning and reperfusion injury. Nat Rev Cardiol. (2016) 13:193Cardio 10.1038/nrcardio.2016.5 26843289

[70] Ezzati M Bainbridge A Broad KD Kawano G Oliver-Taylor A Rocha-Ferreira E. Immediate remote ischemic postconditioning after hypoxia ischemia in piglets protects cerebral white matter but not grey matter. J Cereb Blood Flow Metab. (2016) 36:1396–396:6 10.1177/0271678X15608862 26661194 PMC4976661

[71] Lim SY Hausenloy DJ. Remote ischemic conditioning: from bench to bedside. Front Physiol. (2012) 3:27. 10.3389/fphys.2012.00027 22363297 PMC3282534

[72] Munk K Andersen NH Schmidt MR Nielsen SS Terkelsen CJ Sloth E. Remote ischemic conditioning in patients with myocardial infarction treated with primary angioplasty: impact on left ventricular function assessed by comprehensive echocardiography and gated single-photon emission CT. Circ Cardiovasc Imaging. (2010) 3:656gsc I 10.1161/CIRCIMAGING.110.957340 20826592

[73] Botker HE Kharbanda R Schmidt MR Bottcher M Kaltoft AK Terkelsen CJ. Remote ischaemic conditioning before hospital admission, as a complement to angioplasty, and effect on myocardial salvage in patients with acute myocardial infarction: a randomised trial. Lancet. (2010) 375:727d tri 10.1016/S0140-6736(09)62001-8 20189026

[74] Wang ZK Liu FF Wang Y Jiang XM Yu XF. Let-7a gene knockdown protects against cerebral ischemia/reperfusion injury. Neural Regen Res. (2016) 11:262sgen 10.4103/1673-5374.177734 27073379 PMC4810990

[75] Hong H Tao T Chen S Liang C Qiu Y Zhou Y. MicroRNA-143 promotes cardiac ischemia-mediated mitochondrial impairment by the inhibition of protein kinase Cepsilon. Basic Res Cardiol. (2017) 112:60. 10.1007/s00395-017-0649-7 28887629

[76] Gustafsson AB Gottlieb RA. Heart mitochondria: gates of life and death. Cardiovasc Res. (2008) 77:334sc Re 10.1093/cvr/cvm005 18006487

[77] Sun HS Feng ZP. Neuroprotective role of ATP-sensitive potassium channels in cerebral ischemia. Acta Pharmacol Sin. (2013) 34:24ol Si 10.1038/aps.2012.138 23123646 PMC4086509

[78] Ziu M Fletcher L Rana S Jimenez DF Digicaylioglu M. Temporal differences in microRNA expression patterns in astrocytes and neurons after ischemic injury. PLoS ONE. (2011) 6:e14724. 10.1371/journal.pone.0014724 21373187 PMC3044134

[79] Sun C Zhu L Ma R Ren J Wang J Gao S. Astrocytic miR-324-5p is essential for synaptic formation by suppressing the secretion of CCL5 from astrocytes. Cell Death Dis. (2019) 10:141. 10.1038/s41419-019-1329-3 30760705 PMC6374376

